# Docking analysis of phenolic acid and flavonoids with selected TAS2R receptors and in vitro experiment

**DOI:** 10.1038/s41598-024-66861-w

**Published:** 2024-07-10

**Authors:** Oskar Szczepaniak, Maria Jokiel, Kinga Stuper-Szablewska, Joanna Kobus-Cisowska

**Affiliations:** 1 Department of Gastronomy Science and Functional Foods, Poznań University of Life Sciences, ul. Wojska Polskiego 28, 60-637 Poznań, Poland; 2 Department of Biochemistry and Biotechnology, Poznań University of Life Sciences, ul. Dojazd 11, 60-132 Poznań, Poland; 3 Łukasiewicz Research Network – PORT Polish Center for Technology Development, ul. Stabłowicka 147, 54-066, Wrocław, Poland; 4 Department of Chemistry, Poznań University of Life Sciences, ul. Wojska Polskiego 75, 60-625, Poznań, Poland

**Keywords:** *Cornus mas*, Docking calculations, TAS2R, Bitter taste masking, Computational chemistry, Metabolomics, Molecular modelling

## Abstract

Cornelian cherry fruits contain a wide range of phenolic acids, flavonoids, and other secondary metabolites. Selected flavonoids may inhibit the perceiving of bitterness, however, the full mechanism with all TAS2R bitter taste receptors is not known. The aim of the study was to determine the inhibitory effect of *Cornus mas* phenolics against the bitterness receptors TAS2R13 and TAS2R3 through functional in vitro assays and coupling studies. The overall effect was validated by analysing the inhibition of the receptors activity in cells treated with tested cornelian cherry extracts. The strength of interaction with both TAS2R receptors varied between studied compounds with different binding affinity. Most compounds bonded with the TAS2R3 receptor through a long-distant hydrophobic interaction with Trp89A and π–π orbital overlapping—between phenolic and tryptophane aromatic rings. For TAS2R13 observed were various mechanisms of interaction with the compounds. Nonetheless, naringin and quercetin had most similar binding affinity to chloroquine and denatonium—the model agonists for the receptor.

## Introduction

Taste is one of the basic senses used for food tasting. Information sent to the brain from taste receptors (TRCs) is not only limited to the flavors of the food tasted, but also provides the brain with information on the potential. The TRCs are cells in located the taste buds of the mouth and throat. The human body distinguishes five basic tastes (salty, sour, sweet, bitter, and umami). The bitter taste is perceived with the help of 25-layer proteins belonging to the group of bitter taste-sensing type 2 receptors (TAS2Rs), and has been evolutionally connected with signalling spoilt or toxic foods.,. These receptors are classified as a part of the G-protein coupled receptor (GPCR) family and are very important actors in the inflammation state and the immunological cellular response, by activating, proliferating, and targeting cellular migration^1,2^. The group of TAS2R receptors is very diverse in its structure, and affinity to interact with active compounds^3,4^. The TAS2R receptor group displays significant structural diversity, meaning that receptors within this group vary in their molecular configurations. This structural variation allows different TAS2R receptors to interact with a wide range of bitter compounds present in food and beverages. Each TAS2R receptor has a unique shape and chemical makeup, allowing it to recognize and bind to specific bitter molecules with varying degrees of affinity^5^. The varying affinities of TAS2R receptors for interacting with active compounds mean that certain receptors may have a higher or lower sensitivity to specific bitter substances. Some TAS2R receptors may be highly sensitive to certain bitter compounds, while others may have lower sensitivity or may not respond at all to those same compounds^6^. This differential sensitivity contributes to the ability of the body to detect a broad spectrum of bitter tastes. The diversity within the TAS2R receptor group allows the detection of a wide array of bitter substances, ranging from naturally occurring plant compounds to potentially harmful toxins. This broad detection capability enhances the body's ability to identify and avoid potentially harmful or spoiled foods and beverages, thereby aiding in survival and maintaining health. By having a diverse set of TAS2R receptors with varying affinities and specificities, the body can effectively discern a wide range of bitter tastes, providing valuable information about the safety and quality of ingested substances^5^.

Cornelian cherry (*Cornus mas* L.) is rich in numerous flavonoid, iridoid, and anthocyanin compounds, which could act as potent antioxidant and anti-inflammatory agents^7,8^. Active compounds may enhance the viability of cells induced to the source of inflammation or oxidation state, affect the levels of cytokine released and the expression of genes responsible for quenching reactive oxygen species, e.g. SOD-1 and Nox-4^7,9^. Sozański et al.^10^ noted that cornelian cherry fruits significantly increased PPARα protein expression in liver of hyperlidemic rabbits, indicating that the effect may stem from enhanced fatty acid catabolism and helped regulate oxidative stress in the liver and significantly decrease serum levels of IL-6 (A) and TNFα proinflammatory cytokines. Furthermore, our previous study showed that *Cornus mas* fruits a wide range of phenolic compounds that may have synergistic antioxidant effects, as well as affect the decrease in the activity of TAS2R3 and TAS2R13^11^. As our previous study showed insignificant but noticeable effect of TAS2R inhibition, we decided to analyze more cultivars of the plant. As we observed in our research and colleagues' research, different cultivars of one plant may carry diverse functional effects due to the different quantitative and qualitative concentrations of bioactive compounds.

Taking this all into account we formed two hypotheses of this study:

1. The effect of the cornelian cherry fruits on TAS2Rs activity depends on the plant cultivar used and the concentration of phytochemicals in the cultivar studied.
2. The phenolic compounds present in *Corni fructus* may interact with the evaluated TAS2Rs in different loci of the receptor and by other different mechanisms, which affects the overall TAS2Rs activity.

## Results and discussion

**Figure 1:**
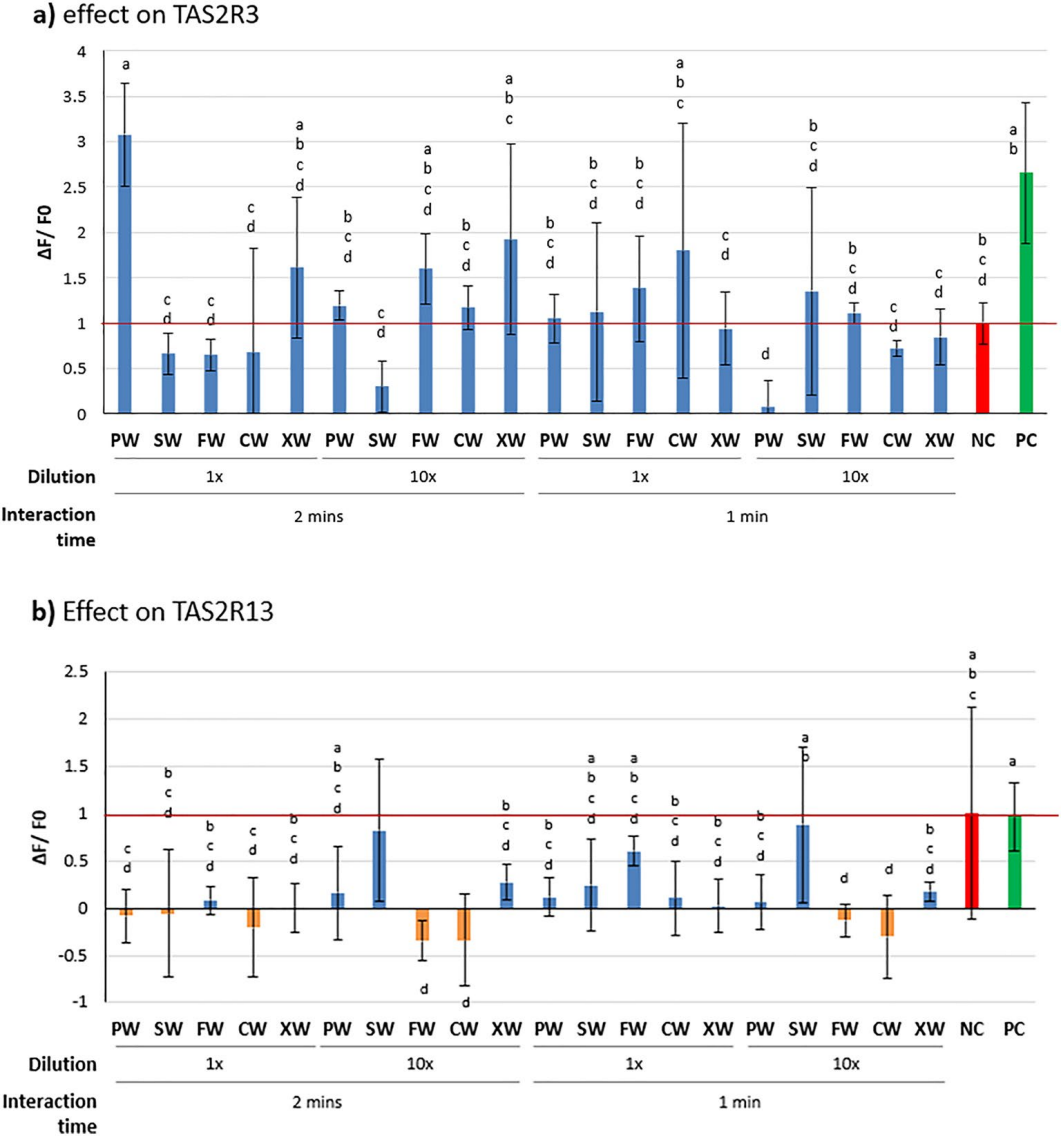
Inhibitory activity of *Cornus mas* extracts on bitter taste receptors: (**a**) TAS2R3; (**b**) TAS2R13. *NC* negative control (PBS buffer), *PC* positive control (30 mM chloroquine for TAS2R3 and 30 mM denatonium for TAS2R13), *CW* aqueous extract of cultivar *Jolico*, *FW* aqueous extract of cv. *Florianka*, *PW* aqueous extract of cv. *P5*, *SW* aqueous extract of cv. *Szafer*, *XW* aqueous extract of cv *Wydubiecki*. The orange bars indicate ΔF/F0 lower than zero. The lowercase superscript letters illustrate significant differences between the samples (α = 0.05).

For the purpose of this study, we analysed the effect of the following *Cornus mas* cultivars: *Jolico* (CW), *Florianka* (FW), P5 (PW), *Szafer* (SW), and *Wydubiecki* (XW). We observed differences in bitter-masking effect between the tested *Corni fructus* cultivars, interaction times, and exposed bitter taste receptors. However, Tukey post-hoc test showed no significant differences between time and dilution, due to high deviation in tested samples (Fig. 1). Such a high variance we also noted in our previous study, where one cornelian cherry cultivar was studied as a potent bitterness masking agent^11^.

The extract constituents of *Corni fructus* possessed different activity against TAS2R3 and TAS2R13 receptors (Fig. 1a). For the first receptor, the vast majority of samples had a similar strong activating effect, which could be linked with perceived intensified bitterness. However, this effect needs further confirmation by checking how the active constituents of *Cornus mas* may interact with TAS2R3 together with chloroquine or other bitter compounds (quinine). The results of future research may provide the missing information how phenolic compounds and other ingredients of cornelian cherry could mask or modulate the bitterness perception during consumption of naturally bitter foods.

The strong effect was observed for 2-min treatment with diluted extracts of *Wydubiecki* and *Florianka* cultivars (samples XW and FW and respectively), which promoted the activity of TAS2R3 30–50% poorer than 30 mM chloroquine—the positive control. For this samples we also noted a positive significant relation between the interaction time, and receptor activity. Surprisingly, the application of raw extracts resulted in similar or lower calcium release of the receptor, except P5 cultivar (PW), which 2-min activity was similar to the activity of the positive control. This may suggest that the receptor may interact with many compounds using different ligand pockets, and the compounds may act as reverse agonists, while in the low-concentration environment the extract constituents rather have a pure agonistic effect. The multi-directional effect of the extract constituents might also lead to higher deviation in calcium release of the receptor, which was observed for the tested samples in this study.

For all cultivars tested, we observed a decrease in the calcium release of the TAS2R13 receptor, except the diluted *Szafer* extract (Fig. 1b). The strongest effect was observed for crude extracts of *P5, Jolico*, and *Wydubiecki* (PW, CW, XW, respectively), which led to over 100% inhibition of the calcium release for TAS2R13. This strongly contrasts with the effects observed in TAS2R3 activity for these samples. Such opposite action for two receptors belonging to one family indicates that both receptors studied can have a different mechanism of interaction with ligands and a feasibly different mechanism of action.

Among the tenfold diluted extracts, *Jolico* and *Florianka* (CW, FW) were found to be the strongest activator of the receptor. Furthermore, the longer exposition time (2 min) gave a poorer masking effect, which may show that the affinity of the active compounds for TAS2R13 is much poorer than for 30 mM denatonium—the positive control.

However, this effect needs to be further confirmed by investigating how the active constituents of *Cornus mas*, together with denatonium, may interact with TAS2R13.

Furthermore, the effects observed for TAS2R13 cannot fully support the defined hypothesis, since the activity of the receptor was similar for both positive (30 mM denatonium solution) and negative controls. Therefore, the potential conclusions from this part of the study are very limited, but the results can be used for comparison in further research.

To verify whether the effects observed in the TAS2R experiment depend on the chemical composition of the extracts tested, we performed a quantitative HPLC assessment of the phenolics present in the extracts tested (Table 1).

**Table 1:** Phenolics characterisation of the tested *Cornus mas* extracts (mg/ml).

Sample	Caffeic acid	Ferulic acid	Kaempferol	Naringin	Quercetin	Vanillic acid	Vitexin	Total phenolic count
PW	6.60^e,f,g^ ± 0.09	24.50^c,d^ ± 1.15	ND	ND	ND	4.80^f,g,h,i^ ± 0.12	2.14^g,h,i,j^ ± 0.76	27.51^c^ ± 3.14
CW	0.80^i,j^ ± 0.02	1.19^h,i,j^ ± 0.02	0.01^j^ ± 0.01	58.48^b^ ± 6.07	0.42^i,j^ ± 0.02	ND	5.41^e,f,g,h^ ± 1.13	20.15^d^ ± 0.42
SW	0.80^i,j^ ± 0.05	1.31^h,i,j^ ± 0.06	0.04^j^ ± 0.01	ND	0.59^i,j^ ± 0.01	0.34^i,j^ ± 0.06	6.38^e,f,g^ ± 0.55	28.55^c^ ± 0.24
FW	1.30^h,i,j^ ± 0.04	1.26^h,i,j^ ± 0.06	0.11^j^ ± 0.01	78.22^a^ ± 4.74	0.42^i,j^ ± 0.01	0.03^j^ ± 0.08	9.51^e^ ± 1.06	24.62^c,d^ ± 0.10
XW	2.20^g,h,i,j^ ± 0.32	1.28^h,i,j^ ± 0.04	0.01^j^ ± 0.01	ND	0.45^i,j^ ± 0.02	0.14^j^ ± 0.01	9.22^e,f^ ± 0.12	22.39^d^ ± 0.56

*ND* not detected. The lowercase superscript letters illustrate significant differences between the samples (α = 0.05).

The differences observed in TASRs inhibition between the tested cultivars of *Cornus mas* should depend on individual binding affinity of the phenolics and not on their quantitative effect. To confirm this hypothesis we selected 7 compounds, for which no significant difference was found between the tested cultivars: caffeic acid, ferulic acid, kaempferol, naringin, quercetin, vanillic acid, and vitexin.

Phenolic compounds can interact with TAS2R receptors in various ways and at different sites on the receptor molecule. These interactions may involve binding to specific regions of the receptor protein, thereby altering its conformation or activity. Additionally, phenolics may influence TAS2R activity through mechanisms such as modulation of intracellular signalling pathways or direct effects on cellular responses. Phenolic compounds can bind to specific binding sites on TAS2R receptors, leading to conformational changes in the receptor structure^6^. This binding can affect the receptor's ability to recognize and respond to bitter compounds, either by enhancing or inhibiting its activity. The interaction between phenolics and TAS2R receptors at these binding sites can modulate the overall sensitivity of the receptor to bitter stimuli. Phenolic compounds may also exert their effects on TAS2R activity through alternative mechanisms. For example, they may influence intracellular signalling pathways involved in bitter taste transduction^5^. By affecting signalling molecules or pathways downstream of TAS2R activation, phenolics can indirectly regulate TAS2R activity and alter taste perception. Phenolics may have direct effects on cellular responses to bitter stimuli, independent of their interaction with TAS2R receptors. These compounds can affect cellular processes such as neurotransmitter release, ion channel activity, or gene expression, which ultimately impact the perception of bitter taste^5^.

The docking study resulted with different binding affinities of the cornelian cherry phenolics and their binding sites (Table 2). We used the confirmed receptor agonists as a reference in our docking study to compare whether the evaluated compounds could interact at the same locus as the agonist. The locus of the possible interaction may help determine whether the docked phenolic may act as antagonist or reverse agonist of TAS2R.

**Table 2:** Characteristics of binding of the tested compounds to the bitter taste receptors.

Receptor	Ligand	Binding affinity (kcal/mol)	Binding fragments	Pocket no
TAS2R3	Chloroquine	− 7.5	**P:** Trp89A, Lys266A, Phe258A, Phe250A, Ile69A; **A:** Trp89A	1
	Caffeic acid	− 6.5	**P:** Leu184A, Trp89A; **A:** Trp89A; **H:** Asp86A; Glu177A, Trp89A	1
	Ferulic acid	− 6.2	**P:** Trp 89A, **A:** Trp89A; **H:** Glu169A, Ser66A	1
		− 6.2	**P:** Phe240A, **A:** Phe240A, Tyr200A; **H:** Ser196A	3
	Kaempferol	− 6.5	**P:** Trp89A; **A:** Trp89A	1
	Naringenin	− 7.3	**P:** Leu184A, Phe250A, Trp89A; **A:** Trp89A; **H:** Trp89A, Glu177A	1
	Quercetin	− 7.5	**P:** Trp89A, Ile69A; **H:** Lys266A, Asn74A, Glu270A, Ser66A, Glu70A	1
	Vanilic acid	− 6.0	**P:** Ile69A; A: Trp89A; **H:** Asn74A, Ser66A	1
	Vitexin	− 6.7	**P:** Ile111A, Leu207A; **H:** Gly287A, Ser289A, Lys110A	2
TAS2R13	Denatonium	− 8.7	**P:** Thr182A,Trp89A, Phe175A, Ile262A; **A:** Trp89A	1
	Caffeic acid	− 6.6	**P:** Ile262A; **H:** Leu156A	1
	Ferulic acid	− 6.5	**P:** Phe175A; **H:** Leu156A	1
	Kaempferol	− 8.6	**P:** Ile262A, Phe175A; **H:** Asp157A, Cys247A	1
	Naringenin	− 8.6	**P:** Trp89A; **A:** Trp89A, Phe175A; **H:** Trp89A	1
	Quercetin	− 8.4	**P:** Ile262A, Phe175A; **H:** Asp157A, Cys247A	1
	Vanilic acid	− 5.7	**P:** Ile262A; **H:** Asn259A, Ser251A, Tyr257A	1
	Vitexin	− 5.4	**P:** Trp252A, **H:** Glu173A, Lys180A, Ser176A, Trp252A	X

*P* hydrophobic pocket, *A* π–π interaction between aromatic rings, *H* hydrogen bond(s), *X* other than predicted based on denatonium structure. Gray shade indicate the positive controls (TAS2Rs agonists) used in the HEK 293-T cell study.

**Figure 2:**
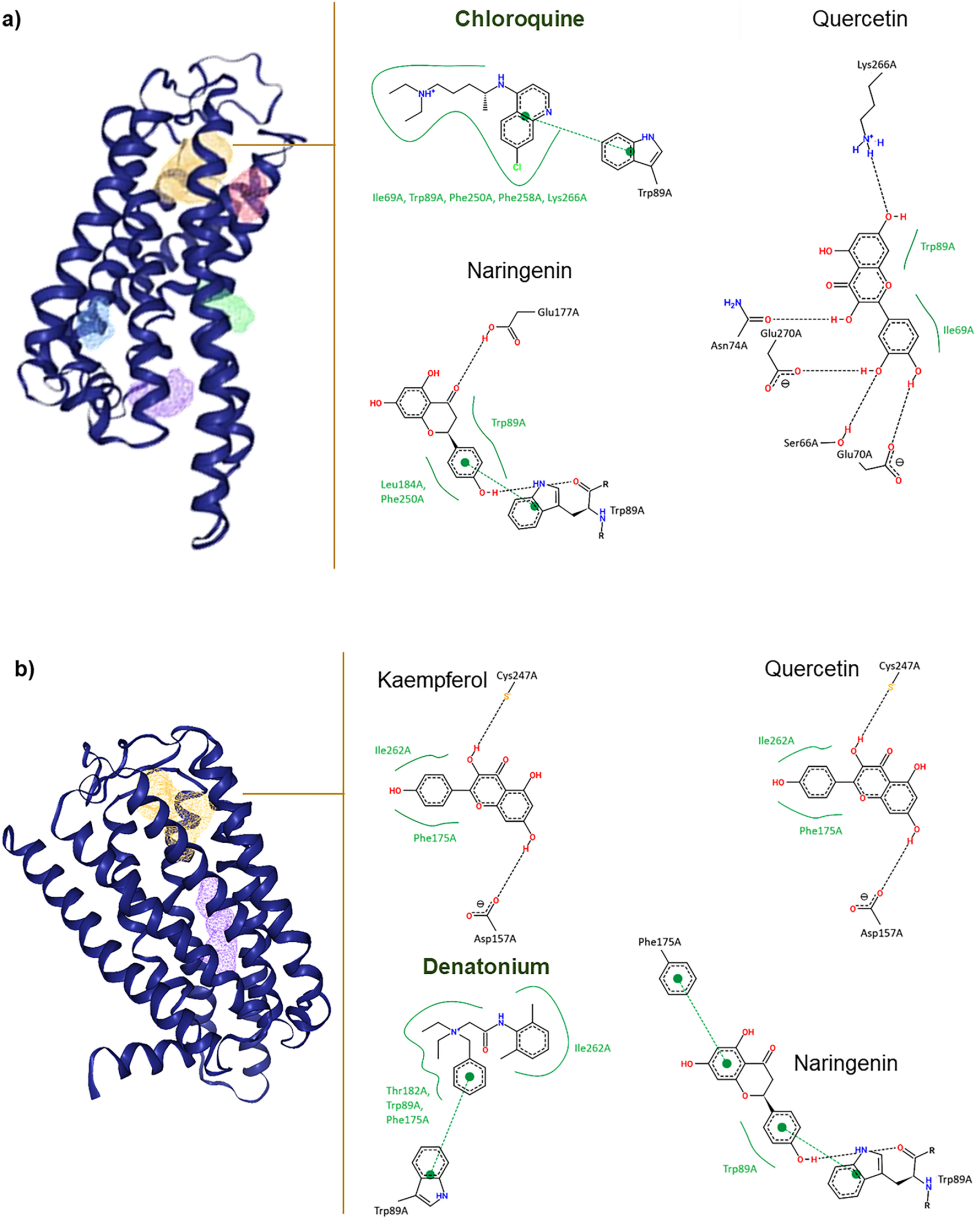
Ligand pockets of TAS2R3 (**a**) and TAS2R13 (**b**). Positive controls are marked with dark green. Based on own data deposited in 10.18150/Q2D4XE and 10.18150/UAN6T2.

Chloroquine—the strongest agonist of TAS2R3 receptor bonds with − 7.3 kcal/mol affinity in a hydrophobic pocket created by a peptide fragment Trp89A, Lys266A, Phe258A, Phe250A and Ile69A (orange cloud in Fig. 2a). The confidence of the pocket structure is prevalently high or very high (pLDDT value over 67), except Phe258A. For this amino acid the confidence equaled 68.7 (see Supplementary material). Chloroquineis additionally stabilised by π-π interactions between its aromatic rings and aromatic ring of Trp89A (Supplementary material available at 10.18150/Q2D4XE). This mechanism was found to be also responsible for interactions between all the tested phenolics, except vitexin.

For TAS2R13, the inhibition mechanisms of phenolics are more diverse and differ noticeably from denatonium (binding affinity − 8.7 kcal/mol) despite the fact that almost all of them uses the same hydrophobic pocket. The control compound binds with the receptor placing a pocket created by Ile262A and Thr182A, Trp89A and Phe175A (orange cloud in Fig. 2b). Additionally, the compound is stabilized by long-distance interaction with Trp89A, analogically to TAS2R3. The predicted mechanism of interaction has confidence ranges from nearly 62 to nearly 90 pLDDT value. However the fragments with lowest confidence take no action with denatonium (see Supplementary material). In spite of similarity in the ligand pocket structure and stabilisation mechanisms between two receptors, most phenolics bind only to a fragment of the denatonium locus, especially to isoleucine or isoleucine and phenylalanine fragment of the pocket, and are additionally stabilized by hydrogen bonds (Supplementary material available at 10.18150/UAN6T2). The strongest affinity to TAS2R13 had kaempferol and naringenin (− 8.6 kcal/mol). The other compound binds to the receptor in place of Trp89A and interacts with the amino acid by both hydrophobic long-distance and hydrogen bonds.

For both receptors, phenolic acids had a low binding affinity. This could result from their small sizes comparing with other tested phenolics, which could cause partial occupation of receptor pocket, and high hydrophilic character which can limit the affinity strength, as pocket-creating amino acids have prevalently hydrophobic character.

**Figure 3:**
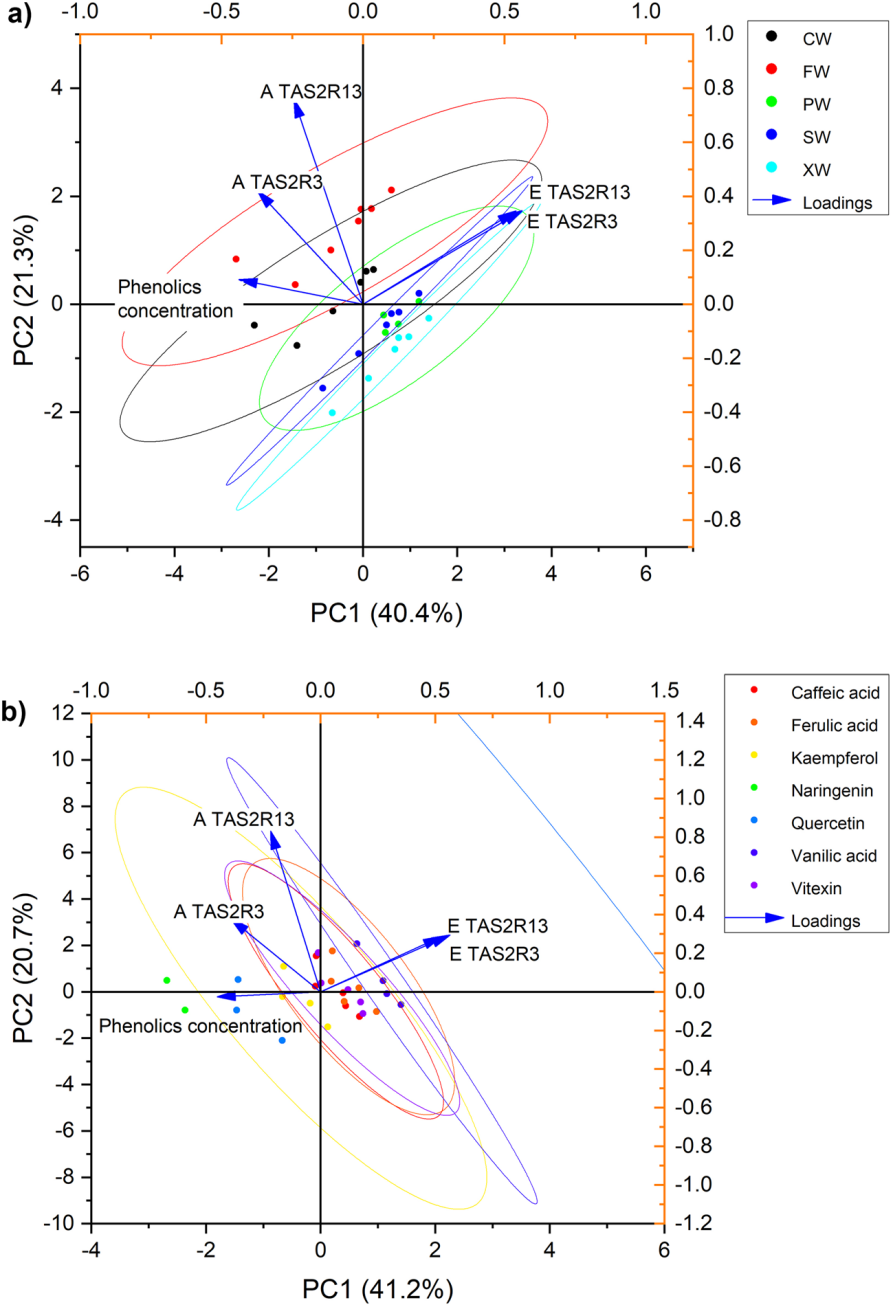
Principal component analysis of the evaluated cornelian cherry cultivars (**a**) and factors (**b**): A TAS2R3—the activity of TAS2R3 after interaction with raw extract by 1 min, A TAS2R13—the activity of TAS2R13 activity after interaction with raw extract by 1 min, E TAS2R3—binding energy to TAS2R3, E TAS2R13—binding energy to TAS2R13. Color ovals represent 95% confidence level.

Principal component analysis showed that tested variables represent over 60% factors affecting the model describing differences between tested samples, and for different compounds (Fig. 3a). PCA showed that cvs. Szafer, P5 and Wydubiecki had bitterness-masking activity as they related negatively with activity of TAS2R receptors. Conversely, cvs. Florianka and Jolico promoted higher activity of the receptors. The high concentration of the phenolics had insignificant but slightly positive effect on the receptors activity and perceived bitterness. Moreover, the relation between two TAS2Rs and the phenolics binding affinity energy is negative, which means that the receptors interaction is exoergic. Apparent is that individual phenolics may bind and affect the perceived bitterness, but the strength of the bitterness inhibition is rather limited. This confirms the results from the HEK 293T study, where the observed effect was noticeable but insignificant.

Visualization of differences for the studied phenolics show that higher concentration of kaempferol and quercetin affected positively TAS2R3 activity, i.e. the bitterness perceived (Fig. 3b). Conversely, phenolic acids evaluated as well as vitexin tended to decrease the activity of the receptor. PCA also showed that binding affinity is negatively related with TAS2R3 activity, due to other pockets where the interaction occurred. Surprisingly, the evaluated phenolics had no effect on TAS2R13 activity despite strong binding affinities. Only a negative relation was found for the extracts used. Plausibly, the activating/inhibitory effect on TAS2R13 might be induced only for low doses of the compounds, as over the limit the associations and non-specific docking to the receptor surface could occur.

Przeor and Jokiel^12^ observed that the conditioning stage affects the content of bioactive compounds in *Morus alba* L., and thus the activity of TAS1R receptors. In the referred study, changes in content of gallic, chlorogenic and caffeic acids resulted in over three-fold higher activity of TAS1R sweet taste receptors, which may be considered as increased perception of the sweet taste. Moreover, the *Morus alba* extract noticeably inhibited the activity of TAS2R13 bitter taste receptor both after 1- and 2-min interaction time.

The effect of plant secondary metabolites on taste perception might be very important in context of consumers’ health. The sensitivity of taste perception is related with expression of genes coding taste receptor and gene polymorphism. The gene polymorphism of TAS2R receptors causes the presence of different receptor proteins, i.a. TAS2R3 (rs765007), TAS2R5 (rs2234012), TAS2R19 (rs10772420) and TAS2R50 (rs1376251). Each receptor from the TAS2R family has different affinity to individual compounds and potential interaction locus, which further results in diverse bitterness perception of the same food product amongconsumers with high genetic deviation. This can eventually affect humans’ nutritional choices and levels of their metabolical markers in blood.

The taste sense and receptors associated with it have still been not completely explored. So far, the function of TAS2R receptor has been examined only in partial. The result of this work shows that bioactive ingredients of cornelian cherry fruits may affect the activity of the bitter taste receptors, which could further influence the perception of different foods enriched in these ingredients. We also simulated the interaction mechanism of *Cornus mas* phenolic compounds with TAS2R receptors, and revealed that most of them locate in the same locus of the receptor as the control compounds, and interacts with at least one amino acid of the active centre analogically as the control. The evaluated phenolics of *Cornus mas* interacted mostly with TAS2R3 by long-distance van der Waals interactions with Trp89A fragment, supported by π-π orbitals overlapping with aromatic rings. Conversely, the interaction mechanism with TAS2R13 differed between the tested phytocompounds. However, naringin and quercetin had the most similar binding affinity and mechanism to denatonium—the strongest agonist of TAS2R13. However for this compounds confirmed was bitterness-promoting levels^13–15^. This suggests that potential TAS2Rs inhibitors or other bitterness-masking agents should be sought among compounds, which interact with the receptors in other locus than denatonium. Such a compound could be vitexin, which tends to interact in different receptor pockets of both studied TAS2Rs.

## Conclusions

Analysis of the extract constituents of *Cornus mas* revealed differing activities against TAS2R3 and TAS2R13 receptors. For TAS2R3, most samples exhibited strong activation of the receptor, potentially intensifying perceived bitterness. The greatest effect was observed with 2-min treatments of diluted extracts from the *Wydubiecki* and *Florianka* cultivars, while the highest TAS2R3 activity was promoted by the raw extract of P5 (even more stronger than the positive control, chloroquine). Interestingly, the application of raw extracts resulted in similar or lower receptor activity, suggesting that different compounds within the extracts may interact with taste receptors using different mechanisms. In low-concentration environments, these compounds may act as pure agonists, while in higher concentrations, they may function as reverse agonists. However, this speculations should be confirmed in further research including co-addition of the extract constituents with the confirmed agonists of the bitter taste.

Among the tenfold diluted extracts, *Jolico* and *Florianka* were identified as the strongest agents on TAS2R13 activity. Interestingly, longer exposure times (2 min) resulted in a poorer masking effect, indicating lower affinity of active compounds to TAS2R13 compared to the positive control, denatonium.

Nonetheless, these results should be taken with precaution since the activity of TAS2R13 was similar for both positive control (30 mM denatonium) and the negative.

To investigate whether observed effects on TAS2R receptors depended on the chemical composition of the extracts, we conducted an HPLC quantitative assessment of phenolics. The differences in TAS2R inhibition among tested *Cornus mas* cultivars were found to be related to individual binding affinities of phenolics rather than their quantitative effects. This was confirmed by selecting seven compounds with no significant differences between cultivars: caffeic acid, ferulic acid, kaempferol, naringin, quercetin, vanillic acid, and vitexin.

In our study, we conducted docking analyses to investigate the binding affinities of phenolic compounds from cornelian cherry to TAS2R taste receptors. We utilized confirmed receptor agonists as reference compounds to assess whether the evaluated phenolics could interact at similar binding sites. Understanding the binding locus is crucial for determining whether the docked phenolics may act as antagonists or reverse agonists of TAS2R receptors.

Results revealed that over 60% of the factors influencing the model were represented by the tested variables. Notably, certain cultivars, namely *Szafer*, *P5,* and *Wydubiecki*, exhibited bitterness-masking properties, correlating negatively with the activity of TAS2R receptors. In contrast, cultivars *Florianka *and *Jolico* were associated with heightened receptor activity. Although high concentrations of phenolics showed a slightly positive effect on receptor activity and perceived bitterness, the overall impact on bitterness inhibition was limited. The relationship between TAS2Rs and phenolics binding affinity energy was found to be negative, indicating an exoergic interaction.

## Materials and methods

### Tested material

Cornelian cherry fruits were collected in full ripeness in the orchard farm Szynsad in Dąbrówka Nowa, Błędów, Mazowieckie, Poland (51°47′01″N 20°43′04″E) on September 2018 at the third year of plants cultivation. The fruits of five cultivars were considered in this study: *Wydubiecki*, *Szafer*, *Jolico*, *Florianka*, and *P5* (encoded as XW, SW, CW, FW and PW). The fruits were stored in refrigerated conditions (cooling temperature = 4 °C) until the extracts were prepared.

The tested extracts were prepared analogically as in our previous study^16^. The fresh fruits were firstly cut so as to facilitate proper maceration of the plant material. Then, fruits were macerated with distilled water at mass/volumetric (m/v) ratio 1:5 for 30 min at 40 °C. After that period, the extracts were filtered through paper filter (Alfachem, Poznań, Poland). The prepared extracts were stored in tubes at − 21 °C until further analysis.

#### Collection statement

Since the plant were collected from a private orchard no special approval or permission was needed. The genetic profile of the cultivars was defined in our previous paper^17^. The person confirming the cultivars used was Prof. Piotr Szulc from Department of Agronomy, Poznań University of Life Sciences, and the person performing the genetic relationship analysis was Prof. Kamila Nowosad from Department of Genetics, Plant Breeding and Seeding of Wroclaw University of Life Science and Environment.

### Preparation of tested cells with taste receptors

We followed o protocol and cell model of HEK-293T (ATCC, USA) cells liposomal transfected with TAS2R3 and TAS2R13 genes described in our previous study^11^.

### Cell transfection

Before the right transfection process we checked the potential of HEK-293T cells to express the transfected genes, using a sequence coding G16-gust44 chimeric gustducine-acting protein cloned into pcDNA 3.1 vector (Sigma-Aldrich, Poland). The expression of the protein was assessed using Western blot technique. The cells with the highest G16-gust44 expression were selected to right part of the experiment.

HEK-293T cells culture, which expressed with highest efficiency G16-gust44 genewere then dissolved to rough concentration of one cell per well in 96-well microplate. Cells were grown in 96 wells microplates (VWR, Poland) at temperature 37 °C, in atmosphere of 5% CO_2_ and 95% air, at humidity level 70%. Cell culture was carried out in DMEM (BIOWEST, United States) medium with additions of inactivated bovine serum (10% vol., Thermo Fisher Scientific, Poland), sodium pyruvate (1% vol., Sigma-Alrich, Poland), Pen-Strep (1% vol., Sigma-Aldrich, Poland) and mixture of essential amino acids (1% vol., Thermo Fisher Scientific, Poland). Microplate with monocell solutions was incubated by 3 weeks to harvest colonies from each singular cell. Then, the cell line was transfected using lypofection method by pcDNA 3.1 plasmids containing genes coding bitter taste receptors (TAS2R13 and TAS2R3). The plasmid vectors were purchased from the local supplier (Thermo Fisher Scientific, Poland).

Overexpression of TAS2R13 and TAS2R3 was confirmed by Western Blot test.

### Taste receptors inhibition test

We based our receptor activity study on fact that any form of agonistic interaction with the receptors tested result in higher Ca^2+^ ions release from the endoplasmatic reticulum to the cytoplasmatic space. Thus, the increased activity of the TAS2Rs and bitterness perceived was considered as higher calcium release.

The cell lines were tested under the release of calcium from the endoplasmatic reticulum in the presence of agonist of separate receptor or in the presence of the tested cornelian cherry extract. Calcium release was determined using Fluo-4 Direct™ Calcium Assay Kit (Thermo Fisher Scientific, Poland), according to the producer’s protocol. Determined was fluorescence 1 min after intake of 5 μl tested extract/reference material onto the cells.

As positive controls were used material 30 mM chlorokin (TAS2R3 bitter taste receptors), and 30 mM denatonium (TAS2R13 bitter taste receptor). As a negative control (NC) a PBS buffer was used.

Final result was presented as ratio between fluorescence growth (ΔF) of cells exposed to the *C. mas* extract and the fluorescence intensity in the control sample (F_0_). The ΔF/F_0_ value lower than 1 was considered as taste receptor inhibition. For each sample and control used we made independent three repetitions.

### Quantitative assay of phenolic compounds and flavonoids

First the extracts were subjected to alkaline and acidic hydrolysis to obtain phenolic aglycons for the chromatographic analysis^18^. The analysis of phenolic compounds was next performed according to the previous method^19^. We used Acquity H class UPLC system equipped with the Waters Acquity PDA detector (Waters, USA) with Acquity UPLC® BEH C18 column (100 mm × 2.1 mm, particle size 1.7 μm) (Waters, Ireland) as the stationary phase. The elution ran in a gradient mode using the following mobile phase composition: A: acetonitrile with 0.1% formic acid, B: 1% aqueous formic acid mixture (pH 2). Concentrations of phenolic compounds were determined using an internal standard at wavelengths λ = 320 nm and 280 nm and finally given at mg/g extract. Compounds were identified based on a comparison of the retention time of the analysed peak with the retention time of standards, Selected compounds had to be analysed using the standard addition method. The detection level for all tested compounds was 1 μg/g. Retention times for phenolic acids were: 15.20 min for caffeic acid, 16.80 min for vanillic acid, 17.50 min for ferulic acid. Retention times for flavonoids were: 8.00 min for vitexin, 11.00 min for kaempferol, 17.00 min for quercetin and 17.50 min for naringenin.

Additionally assessed were total phenolic count to own spectroscopic method^16^.

### Docking analysis

The computational model were prepared of the above-mentioned compounds. The molecular structures well prepared in Gaussview 06 program and optimized using HF/3-21+G* base in Gaussian 16W^20^. After optimization the structures were subjected to docking analysis with models of TAS2R3 and TAS2R13 bitter taste receptors (Uniprot numbers Q9NYW6 and Q9NYV9, respectively). The structures of the tested TAS2Rs were acquired from BitterDB database^4,21^, https://bitterdb.agri.huji.ac.il/dbbitter.php in the form of pdb format. Since the TAS2R models originate from trusted protein repositories, we exempted from optimizing their structures. Ligand pockets of the tested receptors were pre-screened using DocSite3 tool^22^ available at Protein.plus website based on docking of the positive controls: TAS2R13 receptor and chloroquine for TAS2R3, analogically as in the cell-line experiment. The confidence was determined in AlphaFold database in pLDDT scale. As docking tools applied was Autodock Vina software run with Pyrx graphics editor. The docking positions was based on the location of 2–4 potential ligand pockets of the receptors. For TAS2R3 receptor we used the exhaustiveness = 8 and following grid parameters:

$${\text{center}}\_{\text{x }} = \, - 0.0{684521}00{1817};{\text{ center}}\_{\text{y }} = \, - {2}.{6592372589};{\text{ center}}\_{\text{z }} = \, - {3}.{3725}0{253741};$$

$${\text{size}}\_{\text{x }} = { 28}.{8733878674};{\text{ size}}\_{\text{y }} = { 37}.{694874}00{58};{\text{ and size}}\_{\text{z }} = { 49}.{9174274359}.$$

For TAS2R13 receptor, applied was exhaustiveness = 8 and grid parameter, as follows: center_x = 1.43169255417; center_y = − 8.23687720474; center_z = − 7.08160207511; size_x = 55.5253346961; size_y = 72.1749730773; and size_z = 80.9882696374. The coordinates of the docked compounds were saved. 1–2 models of the docked ligands with highest binding affinities were tested to receive their interaction diagrams with fragments of the tested receptors. This stage was done using PoseView^23^ program running on Protein.plus website and resulted in graphical illustrations of amino acids residues participating interacting with ligands. All detailed information and visualisation of the interactions between the tested ligands can be found in the Supplementary material deposited in RepOD database 10.18150/UAN6T2 (TAS2R13) and 10.18150/Q2D4XE (TAS2R3).

### Statistical analysis

For HPLC study we performed one-way analysis of variance (ANOVA) and Tukey post-hoc test to visualise significant differences between the tested cultivars. For the bitter taste receptor study we applied three-way ANOVA to illustrate significant differences between interaction time, cultivar used and dilution of the extract. Normalized and non-normalized results were analyzed separately. The principal component analysis (PCA) was used to present statistical relations between the cultivars used, individual phenolics content, their binding affinity to the taste receptors and results of the cell model study. For all formal analyses Origin Pro 2023b (Origin, Germany) was used.

### Supplementary Information

Supplementary Information.

## Data Availability

The raw data of phytochemicals interaction, the molecule files, and graphical presentation of interaction loci in the tested TAS2Rs will be available at Poznań University of Life Sciences open data repository in two dedicated collections: TAS2R3-interaction; TAS2R13-interaction: 10.18150/UAN6T2 (TAS2R13) and 10.18150/Q2D4XE (TAS2R3). The additional summary of ligand pockets and their confidence has been attached as supplementary file to this article. All other data will be available from the corresponding author on request.
